# Lymphadenectomy before and after radical cystectomy: does this affect the radicality? A prospective randomized comparative study

**DOI:** 10.1007/s11255-023-03826-4

**Published:** 2023-10-17

**Authors:** Ahmed M. Moeen, Diaa A. Hameed, Mohamed G. Mostafa, Shimaa H. Shaban

**Affiliations:** 1 Urology Department, Assiut University, Asyut, Egypt; 2 Pathology Department, Assiut University, Asyut, Egypt

**Keywords:** Urinary diversion, Radical cystectomy, Lymphadenectomy, Bladder cancer

## Abstract

**Purpose:**

To compare the oncological outcome of performing ePLND before or after RC in 200 patients in a prospective randomized manner.

**Materials and methods:**

From January 2014 to December 2019, 200 patients with T2-T3b N0M0 BCa were included in the current study after signing an informed consent. Patients were divided into two groups, 100 in each one. Group I underwent ePLND before RC, whereas group II underwent ePLND after RC. Postoperative evaluation included clinical, laboratory, and radiographic studies.

**Results:**

Patients’ characteristics were comparable between both groups. The mean operative time excluding that of urinary diversion was significantly shorter in group II than in group I (*p* = 0.01). The mean number of LNs removed was 25 ± 6 in group I and 32 ± 8 in group II (*p* = 0.141). Intraoperative complications occurred in four patients in the form of external iliac artery and vein injury [two in each group (*p* = 0. 245)]. Postoperative complications were comparable between both groups with no statistically significant difference (*p* = 0.375). Oncological failure occurred in 28 patients [16 (17.6%) in group I and 12 (22%) in group II (*p* = 0.389)].

**Conclusions:**

EPLND before and after RC has comparable oncological outcomes. The stage of the disease, the time since the first diagnosis till RC and the surgeon experience in performing meticulous ePLND are more important. In absence of oncological superiority, the timing of ePLND should be judged according to the patient-related factors to facilitate safe RC with minimal morbidity.

## Introduction

Neo-adjuvant chemotherapy and radical cystectomy (RC) is the standard management for muscle invasive bladder cancer (BCa) or non-muscle invasive BCa failing intravesical BCG [1]. Pelvic lymph node dissection (PLND) is an essential and crucial step during RC. Different levels of PLND were described with different prognostic outcomes. However, the extended (ePLND) limit is the preferred one. The latter provides more information about staging, prognosis and with better therapeutic benefits [2, 3].

The details of PLND during RC including the limits, precautions, the outcome, the prognostic benefits and the complications are all well described [4–6]. However, little attention was given in the urological literature comparing the effect of timing of PLND on oncological outcomes if the LNs are removed en-block with the bladder or after its removal. Logically, interruption of the lymphatic channels may increase the recurrence rate due to the possible spillage of the malignant cells between the cancerous bladder and the draining lymph nodes (LN). However, there is no clear consensus about this important point in the urological literature.

We hypothesized that, en-block PLND with the RC specimen may be oncologically better, due to lack of interruption of lymphatic channels between the bladder and the draining LNs, than after bladder removal. Thus, the present study was conducted to compare the oncological outcome of performing ePLND before or after RC in 200 patients in a prospective randomized manner.

### Patients and methods

From January 2014 to December 2019, out of 243 patients indicated for RC, 200 patients with T2-T3b N0M0 BCa were included in the current study after signing an informed consent. Patients indicated for palliative cystectomy, those with grossly enlarged LNS in MSCT or MRI, those with chronic kidney disease and who refused to participate in the study were excluded. This study was approved by our institutional ethical committee.

### Preoperative evaluation and randomization

A through preoperative patients’ evaluation including full laboratory workup, abdominal ultrasonography (US), and enhanced CT scan or MRI were done. Patients were divided into two groups, 100 in each one. Group I underwent ePLND before RC, whereas group II underwent ePLND after RC. Randomization was performed using random numbers generated by computer software (JMP, Version 12.0.1; SAS Institute, Cary, NC, USA). The timing of ePLND was revealed to the operator on the day of the surgery via a sealed envelope.

Patient’s preoperative data, the operative time, intraoperative complications, and the postoperative data were analyzed. The latter included the time to oral feeding, drains and ureteric stents removal, time to discharge and the reason for readmission if present. The modified Clavien–Dindo system was used to grade the perioperative complications [7].

### Outcome measures

The primary end point of the current study is to compare the effect of timing of ePLND before or after RC on the oncological outcome. The secondary endpoints are; the difference in the operative time, morbidity of ePLND, and the lymph nodes yield between the studied groups.

### Intervention

RC with ePLND was performed in all patients followed by construction of the suitable urinary diversion (UD) technique (Table 1). All lymphatic tissues around the common iliac artery proximally to the circumflex iliac vein distally, and from the genitofemoral nerve laterally to the bladder medially including the obturator group were removed. Combined antegrade and retrograde RC was performed [2]. Surgical Clips or cauterization were used to control the lymphatic vessels. Two wide-bore drainage tubes were placed in the pelvis before anatomical closure of the abdomen. The LNs were labelled and sent fresh to pathological examination in separate containers. Two experienced pathologists in BCa were assigned to examine the LNS to improve the results. Un-sectioned LNs were not immersed in fixative and care was taken to make thin slices of the node to ensure optimal penetration of fixative. Serial sectioning at 2 mm intervals perpendicular to the long axis of the LNs was done.

**Table 1:** Patients’ characteristics

	Group I (No. 83)	Group II (No. 86)	P value
Mean age (SD) [years]	58.21 (6.9)	55.03 (7.6)	0.235
Sex			0.326
Male	66 (79.5%)	65 (75.5%)	
Female	17 (20.5%)	21(24.5%)	
Mean BMI (SD)	25.72 (0.34)	24.82 (0.45)	0.459
Smoking History	28 (33.7%)	31 (36.04%)	0.247
Associated comorbidities			0.853
DM	13 (15.6%)	16 (18.6%)	
HTN	18 (21.6%)	17 (19.7%)	
Cardiac problems	12 (14.5%)	11 (12.7%)	
Preoperative histopathology			0.295
TCC	63 (75.9%)	68 (79.1%)	
SCC	14 (28.9%)	11 (26.8%)	
Adenocarcinoma	2 (2.4%)	5 (5.8%)	
Micropapillary variant	3 (3.6%)	2 (2.3%)	
Sarcomatoid variant	1 (1.2%)	–	
Stage			0.513
T2	50 (60.3%)	52 (60.5%)	
T3a	28 (33.7%)	30 (34.9%)	
T3b	5 (6%)	4 (4. 6%)	
Grade			
III	83 (100%)	86 (100%)	0.560
N stage			0.111
N0	70 (84.3%)	68 (79.1%)	
N1	13 (15.7%)	18 (20.9%)	
Associated CIS	23 (27.7%)	19 (22.1%)	0.118
Neo-adjuvant chemotherapy	63 (75.9%)	61 (70.9%)	0.114
Mean number of LNs removed (SD)	28 (6)	32 (8)	0.141
No. of patients with positive LNs	13 (15.6%)	17 (19.7%)	0.112
Operative time of RC and PLND (min)	120 ± 25	105 ± 22	0.01
Estimated blood loss	700 ± 150	650 ± 180	0.314
Median hospital stay in days (range)	14 (12–21)	13 (12–25)	0.516
Type of urinary diversion			0.454
Neobladder	28 (37.3%)	30 (34.9%)	
Ileal conduit	42 (46.9%)	37 (43.02%)	
Single stoma uretero-cutanoustomy	10 (12%)	13 (15.11%)	
Uretero-sigmoidostomy	3 (3.6)	6 (6.9%)	
Creatinine (Mean ± SD) [mg/dl] after 1 year	1.20 (0.06)	1.48 (0.11)	0.113
PH after one year	7.35 (0.019)	7.34 (0.023)	0.811
Bicarbonate (mmol/L) after 1 year	21.36 (1.43)	22.38 (1.41)	0.579
Oncologic failure, No (%), pattern	16 (17.6%)	12 (22%)	0.389
Pelvic mass	4	3	
Pelvic LNs recurrence	4	3	
Pulmonary	2	4	
Colonic	5	2	
Bone	1	–	

### Postoperative care, evaluation and follow-up

Parenteral nutrition was maintained for 2 days, and then oral fluids were introduced. Semisolids were gradually introduced after passing flatus and in presence of good intestinal sounds. Drains were removed once fluid drainage stopped. Subcutaneous heparin was administered in the arms to decrease the incidence of postoperative lymphorrhea. In case of prolonged leakage, creatinine level was checked to differentiate the urinary leakage and lymphorrhea. After stents removal, abdominal US was performed to evaluate the upper urinary tract (UUT).

Our centre routine follow up visits were scheduled monthly/first three months, then every three months/two years and biannually thereafter. Each visit included clinical, laboratory, UUT assessment by abdominal US and diversion-specific evaluation as stomal assessment and continence evaluation. Oncological outcome was assessed by an enhanced abdomino-pelvic CT or MRI after 3 months and biannually thereafter.

### Statistical analysis

Statistical analysis was done using the Statistical Package for Social Sciences (IBM SPSS Statistics, version 22.0, release 22.0.0.0; IBM Corp). Student’s t-test or Mann–Whitney U test was used to compare the two groups for continuous variables and the chi-square test was used for categorical variables. *P* value < 0.05 was considered statistically significant.

## Results

**Fig. 1:**
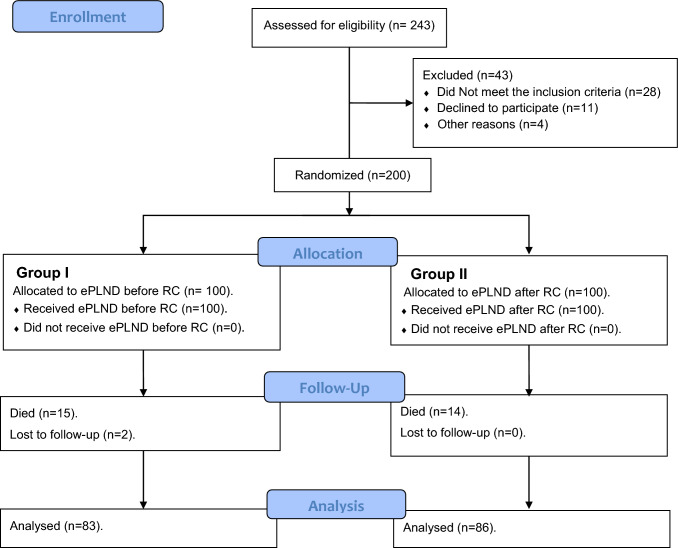
CONSORT flow chart for patients undergoing ePLND before RC (Group I) and after RC (Group II)

During the study period, 200 patients underwent RC and UD for muscle-invasive BCa, 100 in each group. At the last follow up, 169 patients [83 patients in group I and 86 in group II] were analyzed for the final outcome. The remaining 31 patients, 29 died [15 and 14 in both groups, respectively], and 2 patients lost the follow up in group I [Fig. 1]. The preoperative and the discharge laboratory values were within normal range. The causes of death were oncological failure in 20 patients and unrelated medical condition in 9. Patients’ characteristics were comparable between both groups with no statistically significant difference (Table 1).

The mean operative time excluding that of urinary diversion was significantly shorter in group II than in group I (*p* < 0.001) (Table 2). The mean number of LNs removed was 25 ± 6 in group I and 32 ± 8 in group II (*p* = 0.141). The shorter operative time and the increased LN yield in group II may be due to the wider operative field after bladder removal with no need to frequently reposition the surgical retractors and the wide and easy exposure of the pre-sacral group of LNS.

**Table 2:** Postoperative complications

	CDC	Group I (No. 83)	Group II (No. 86)	Total (No, %)	P value
Early postoperative complications					0.375
Blood transfusion	I	63 (75.9%)	65 (75.5%)	128 (75.7%)	
Postoperative fever	I	13 (15.6%)	9 (10.46%)	22 (13%)	
Ileus	I	9 (19.8%)	7 (8.13%)	16 (9.5%)	
Pulmonary embolism	I	2 (2.4%)	1(1.16%)	3 (1.8%)	
DVT	I	5 (6%)	7 (8.13%)	12 (7.1%)	
Urinary leakage	I	5 (6%)	2 (2.3%)	7 (4.1%)	
Wound dehiscence	I	3 (3.6)	4 (4.6%)	7 (4.1%)	
Burst abdomen	IIIa	5 (6%)	3 (3.5%)	8 (4.7%)	
Intestinal fistula	IIIb	1 (1.2%)	–	1 (0.6%)	
Delayed postoperative complications					0.485
Lymphorrhea	8 (9.6%)		10 (11.6%)	18 (10.6%)	
Lymphocele formation	–		1 (13.2%)	1 (0.6%)	
Stomal stenosis	3 (3.6)		2 (2.3%)	5 (2.9%)	
Stomal retraction	4 (4.8%)		1 (1.16%)	5 (2.9%)	
Skin excoriation	9 (19.8%)		10 (11.6%)	19 (11.2%)	
UUT obstruction	7 (8.4%)		5 (5.8%)	12 (7.1%)	
Unilateral	4 (4.8%)		3 (3.5%)	7 (4.1%)	
Bilateral	3 (3.6)		2 (2.3%)	5 (2.9)	
Early postoperative death	2 (2.4%)		1 (1.16%)	3 (1.8%)	

*CDC* Clavien-Dindo classification

Intraoperative complications occurred in four patients in the form of external iliac artery and vein injury [two in each group (*p* = 0. 245)]. Postoperative complications were comparable between both groups with no statistically significant difference (*p* = 0.375). (Table 1) Prolonged TPN was not required in either group with early institution of oral diet. This may be due to preoperative medical optimization, performance of surgery by one experienced surgeon in UD and the application of ERAS with an early postoperative introduction of oral fluids after 2 days in presence of good intestinal sounds even if the patients did not pass flatus.

The length of hospital stay was comparable in both groups (*p* = 0.516). Thirteen patients were readmitted after discharge [8 (9.6%) in group I and 5 (5.8%) in group II (*p* = 0.462)]. The reasons of readmission were hyperchloremic metabolic acidosis with pre-renal increased serum creatinine in 5 patients [3 (3.6%) in group I and 2 (2.3%) in group II] and burst abdomen in 8 patient, [5 (6%) in group I and 3 (3.5%) in group II]. Patients with hyperchloremic metabolic acidosis were managed by IV fluids and sodium bicarbonate and encouraging good oral intake. Patients with burst abdomen underwent wound debridement and closure under spinal anaesthesia.

**Fig. 2:**
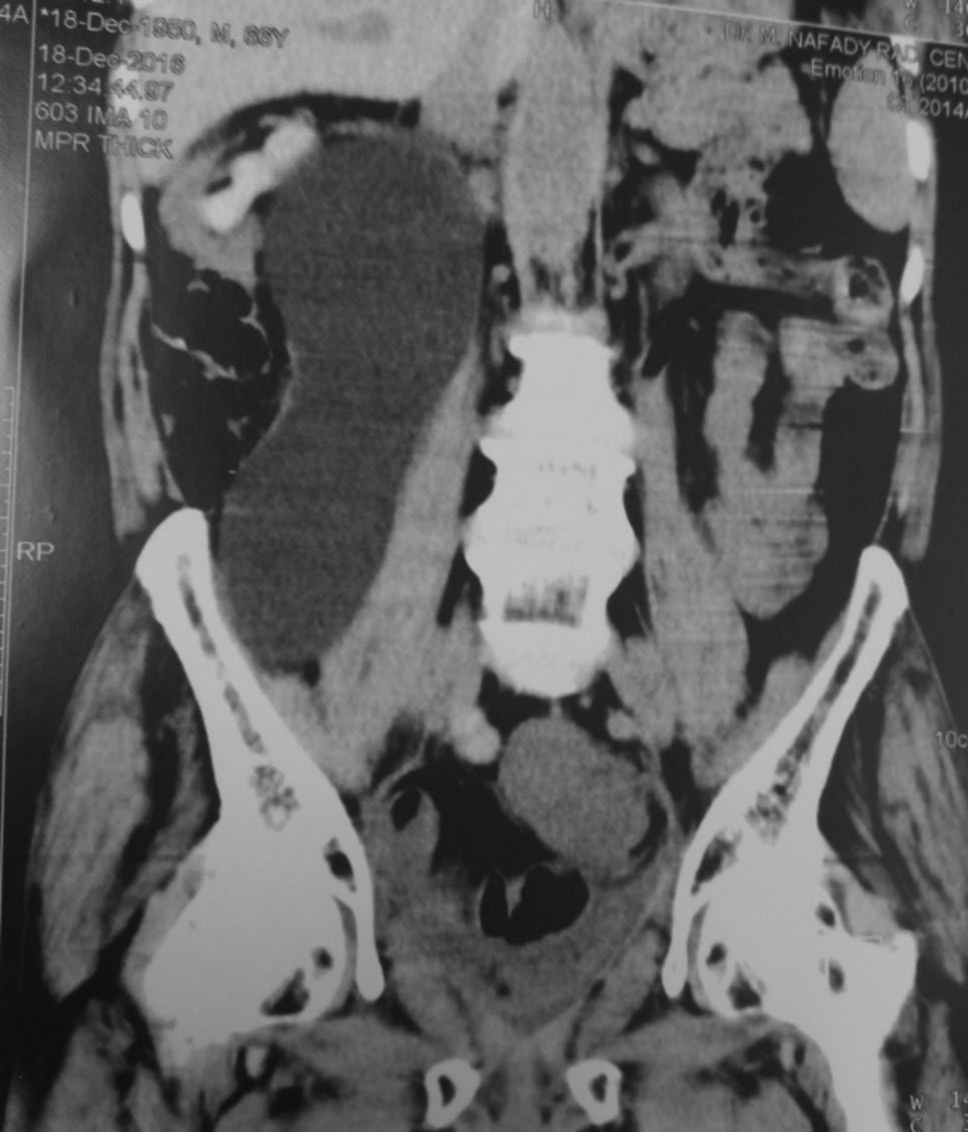
RT sided large lymphocele

Ureteric stents were removed 10–12 day postoperatively in all patients except those with single stoma uretero-cutanoustomy in whom they were exchanged monthly. The ureteric stents and drains were prolonged in 25 patients [18 due to lymphorrhea and 7 due to prolonged urinary leakage (*p* = 0.865)]. Lymphorrhea and urinary leakage were differentiated by double checking the creatinine level and managed by prolongation of drains only. Right sided large lymphocele occurred in one patient in group II with no subsequent effect [no pain or fever], so conservation under observation was enough as recommended by the patient [Fig. 2]. Febrile UTI occurred in 22 patients [10 (12%) in GI and 12 (13.9%) in GII (*p* = 0.625). Those patients were controlled by oral antibiotic only with no need for ancillary procedure.

Oncological failure occurred in 28 patients [16 (17.6%) in group I and 12 (22%) in group II (*p* = 0.389)]. Local pelvic recurrence, [lymph node and local pelvic recurrent mass], each occurred in seven patients in both groups. Distant metastasis occurred in 14 patients [8 (9.6%) in group I and 6 (6.9%) in group II]. The histopathology of the patients in whom oncologic failure occurred was: TCC in 14, moderately differentiated SCC in 6, poorly differentiated SCC in 4 patients, micro-papillary urothelial carcinoma in two and adenocarcinoma in two patients. The pathological tumour stage was T2 in 11 patients, T3a in 9, T3b in 5, and T4a in 3.

## Discussion

Nodal involvement in BCa is associated with a worse oncological outcome. It is reported in 25% of patients. So, it is an important prognostic parameter with a major impact on the outcome and the overall survival. Therefore, careful PLND is critical to stage patients with BCa accurately. The value of PLND has been addressed in details in multiple studies [1–6].

Multiple factors ensure safe and adequate PLND. This includes; the extent of PLND, a meticulous surgical technique, the number of LNs removed including the positive ones, the surgeon and the pathologist experience [2]. However, the effect of timing of PLND, whether before or after bladder removal, on the oncologic outcome of RC was not discussed before, which is the subject of the current study.

The initial site of lymphatic drainage from a primary tumour is called the sentinel node. In BCa, the endopelvic LNs are the sentinel region, metastasis occurs in an orderly fashion and skip lesions are rare. Moreover, crossover lymphatic drainage is seen in 40% of patients. So, PLND should always be performed bilaterally [8–10]. Three levels of PLND in BCa are well described. A limited one involves all LNs in the obturator fossa. Extended (e) PLND includes, the LNs between the aortic bifurcation and common iliac vessels (proximally), the circumflex iliac vein and LN of Cloquet (distally), the genitofemoral nerve (laterally), and the internal iliac vessels (posteriorly), including the obturator fossa and the presacral LNs anterior to the sacral promontory. Super-extended PLND extend to the level of the inferior mesenteric artery. The latter is indicated if frozen section is not performed or identifies positive nodes [11, 12].

The ePLND provides a better local control. It removes about 90% of the primary lymphatic landing sites while the limited one removes only 50%. But, the rate of nodal involvement may be the same. The super-extended PLND increases the number of LNs and the positive nodes removed. However, it has no additional survival benefits, with an increased operative time and patient morbidity [13]. Higher disease stage [T3/T4] and common iliac LN involvement increase the incidence of LN metastasis proximal to the standard PLND in 16% and presacral LNs in 30%, respectively [14]. In our study, the ePLND was done in both groups with good local and distant control. The oncological failure occurred in 28/169 patients (16.5%) [16 (17.6%) in group I and 12 (22%) in group II (p 0.311)], *p* = 0.503]. Local pelvic recurrence; the lymph node and recurrent pelvic mass, each occurred in seven patients in both groups. One patient in our study experienced an early pelvic mass recurrence < 6 months postoperatively in group I. This was despite the early RC after presentation, neo-adjuvant chemotherapy administration and performing the surgery by an experienced urologist. This signifies the aggressive nature of the disease in some patients despite the over-mentioned precautions.

The number of LNs removed and the concept of LNs density are useful prognostic indicators in both LN positive and LN negative patients [15]. A cut-off of 15–20 LNs was considered sufficient for meticulous evaluation of LN status as well as the overall survival. [16, 17]. A 10-year recurrence-free survival of 43% versus 17% in patients with LN densities of ≤ 20% and > 20%, respectively was reported [6, 18]. In our study, the mean number of LNs removed in group I and in group II was 25 ± 6 and 32 ± 8, respectively (*p* = 0.141). However, the number of patients with positive nodes was 13 (15.6%) in group I and 17 (19.7%) in group II (p 0.112). The number of LNs removed in our study was accepted for good PLND as reported [16, 17]. The increased number in group II may be due to the wider field and the easy dissection of the presacral region after RC. So, the presacral area should be evaluated in patients who had ePLND before RC. The easy mobilization of the sigmoid mesentery after RC and the ureters which have been already mobilized and cut facilitates presacral lymphadenectomy.

Nodal involvement is associated with a worse oncological outcome, and may benefit from adjuvant systemic therapies. Its incidence is ranging from 5% in NMIBC, 18–27% in MIBC, 45% in extra-vesical tumours. About 60% of patients with MIBC and LN metastases will die from cancer [19]. The expression of some molecular markers may be useful indicator for the presence of positive nodes as cytokeratin 20 (CK-20), uroplakin II (UP II), mucin 2 (MUC2), and mucin 7 (MUC7). However, their expression may be present but not all LNs are pathologically positive [20].

Performing ePLND before RC may be advantageous as it clearly visualize the vascular pedicles and clarify the tissue planes especially in obese patients. So, RC can be performed more rapidly and without a significant blood loss [21]. However, in presence of large bladder tumours, locally advanced tumours, after previous pelvic surgery and after tri-modality, ePLND before RC is so difficult due to narrow pelvic space and marked pelvic adhesion. Consequently, after bladder removal, the surgical filed will be wider and the dissection more rapid and easier, as the blood and lymphatic vessels are clearly identified. So, in absence of any oncological superiority of PLND whether before or after bladder removal as proved in our study, the surgeon experience and his preference according to the over mentioned patients’ factors are more important to achieve safe outcome.

In a study evaluating the timing of PLND in 2 groups regarding the operative time, the mean ePLND time was similar in both groups (*p* = 0.160). However, the mean RC time and mean total operation times were significantly shorter in group 1 than in group 2 (*p* < 0.001). They recommended that ePLND to be performed before RC because the total operation time is shorter than when ePLND is performed after RC [21]. On the contrary, in our study, the mean operative time in group I was longer than in group II [120 ± 25 min vs 105 ± 22 min, respectively (*p* = 0.01)]. Our results may be due to the wider operative field after bladder removal.

The surgeon experience in performing meticulous PLND can cure 30% of patients with only few LN metastases or with microscopic LN involvement. It was shown that, negative surgical margins and ≥ 10 LN removed were associated with better overall survival independent of patient age, pathological stage, nodal status and neo-adjuvant chemotherapy, all this is surgeon dependent [16]. In addition, an early complication rate of RC of 28% and perioperative mortality rates of up to 3% have been reported [18, 22]. EPLND may prolong operative time by about 60 min. However, this was not associated with an increased morbidity as reported in multiple studies including our study [22–24]. So, it is safe in experienced hands and improves the oncological outcomes by decreasing positive surgical margins and resection of undetected micro-metastases [25, 26].

The Limitations of the current study are, the lack of stage to stage comparison regarding the oncological failure and absence of molecular markers to predict LN positivity. However, the current study is the first one to discuss the effect of timing of ePLND on the oncological outcome of RC in prospective nature over a good cohort of patients, which is a point of strength if compared to the previous LND studies which were retrospective in nature with their inherent biases.

In conclusion, the results of the current study support that lymphadenectomy before and after RC will not compromise the radicality of surgery with similar oncological outcome. However, lymphadenectomy after RC is performed more easily and in a shorter time. The stage of the disease, the time since the first diagnosis of BCa till RC and the surgeon experience in performing meticulous PLND are more important. Moreover, the timing of ePLND should be judged according to the patient’s related factors to facilitate safe RC with minimal morbidity.

## Abbreviations

RC: Radical cystectomy
Bca: Bladder cancer
BCG: Bacillus-Calmette-Guerin
PLND: Pelvic lymph node dissection
(E) PLND: Extended pelvic lymph node dissection
LN: Lymph node
MSCT: Multi-slice computed tomography
MRI: Magnetic resonance imaging
US: Ultrasound
UUT: Upper urinary tract
NMIBC: Non-muscle invasive bladder cancer
MIBC: Muscle invasive bladder cancer
